# Facial Skincare Adverse Event Atlas and Safety Signals From openFDA Cosmetic Reports: A Disproportionality Analysis

**DOI:** 10.1111/jocd.70712

**Published:** 2026-01-30

**Authors:** Zhe Sun, Huiqiong Xiang, Zhuo Fan

**Affiliations:** ^1^ Department of Dermatology The First Affiliated Hospital of Xi'an Medical University Xi'an Shaanxi China

**Keywords:** adverse events, cosmetovigilance, disproportionality analysis, facial skincare, openFDA, reporting odds ratio

## Abstract

**Objective:**

To construct a facial‐skincare adverse event atlas and detect category‐level safety signals using the US FDA openFDA Cosmetic Adverse Events database.

**Methods:**

We analyzed publicly available openFDA cosmetic adverse event reports (downloaded 2025‐12‐15; meta.last_updated 2025‐12‐10). Suspect products were classified into 12 facial‐skincare subcategories using keyword rules, excluding hair, makeup, nail, fragrance, oral, deodorant, and bath/body cleansing products. MedDRA preferred terms were grouped into nine clinically interpretable reaction clusters. Reporting odds ratios (RORs) with 95% confidence intervals were computed versus the full database (primary) and within‐cohort comparators (sensitivity), with false discovery rate (FDR) control. We performed “case‐check” analyses for five prioritized signals to assess product concentration and temporal patterns.

**Results:**

The broad facial cohort comprised 2927 reports and the strict facial sensitivity cohort 1573 reports. The strongest full‐database signals were eye‐area products with ocular symptoms (ROR 61.67; 95% CI 46.13–82.43), masks with burn‐related events (ROR 27.95; 20.39–38.31), retinoid/anti‐aging with swelling/angioedema (ROR 21.94; 17.20–28.00), serum/essence with ocular symptoms (ROR 17.82; 13.74–23.10), and exfoliant/peel/scrub with burn‐related events (ROR 13.39; 9.68–18.53). Within‐cohort comparators attenuated effect sizes but preserved core signals (e.g., eye‐area × ocular symptoms within‐broad ROR 9.14; 6.68–12.49). Case‐check analyses showed low product‐name concentration across signals, supporting category‐level interpretation.

**Conclusions:**

openFDA cosmetovigilance data can be leveraged to map facial‐skincare adverse event patterns and prioritize plausible safety signals. Eye‐area products showed especially strong ocular‐related signals, and leave‐on products (masks, retinoid/anti‐aging, serum/essence) showed coherent burn‐ and swelling‐related signals.

## 1 Introduction

Post‐market monitoring of cosmetic and skincare adverse events (“cosmetovigilance”) is essential for identifying unexpected harms and informing safer product use [1, 2]. Publicly accessible resources such as the US Food and Drug Administration (FDA) openFDA Cosmetic Adverse Events database provide an opportunity to quantify reporting patterns and generate hypotheses about category‐level safety signals [3, 4]. Prior analyses of the FDA's CAERS have described overall reporting trends across product classes, including time‐varying spikes associated with specific product lines and media attention, highlighting the potential for stimulated reporting [5, 6]. CAERS has also been used to summarize adverse event reports related to specific outcomes such as cancer‐related terms [7]. Here, we focus on facial skincare products and develop an adverse event atlas that links product subcategories to clinically interpretable reaction clusters.

## 2 Methods

### 2.1 Data Source and Study Design

We performed a retrospective analysis of the openFDA Cosmetic Adverse Events bulk dataset (downloaded 2025‐12‐15; meta.last_updated 2025‐12‐10) [3, 4]. Reports are spontaneous and may be incomplete or subject to reporting bias; disproportionality metrics reflect reporting patterns rather than incidence or causality.

### 2.2 Database Characteristics

The openFDA cosmetic event download is composed of individual case safety reports identified by a unique report_number. For this download (*n* = 85 511), initial_received_date ranged from 2001‐08‐09 to 2025‐08‐29. Each record contains suspect/concomitant product names, outcomes, patient demographics (when available), and reactions coded as MedDRA preferred terms (MedDRA version 28 in this download).

### 2.3 Cohort Definition and Product Classification

We included reports in which all suspect products were facial‐skincare items and excluded reports with suspect hair, nail, makeup, fragrance, oral, deodorant, and bath/body cleansing products. Only products listed with the role “Suspect” were used for product classification and cohort inclusion. Suspect products were classified into 12 facial‐skincare subcategories using keyword rules. Because some moisturizers and cleansers are multi‐use, we conducted a strict facial sensitivity cohort that required either at least one facial‐specific subcategory (serum/essence, toner/mist, mask, exfoliant/peel/scrub, retinoid/anti‐aging, brightening, sunscreen, acne treatment, eye/area, lip care) or explicit facial cues in product names for moisturizer/cleanser‐only reports while excluding body/hand/foot cues. The strict cohort is a nested subset of the broad cohort.

### 2.4 Reaction Clustering

MedDRA preferred terms in each report were grouped into nine reaction clusters (rash/dermatitis/irritation; pruritus/urticaria/hypersensitivity; burning/chemical/thermal burn; swelling/angioedema; blistering/peeling/dryness; ocular symptoms; pain/paraesthesia; infection; respiratory symptoms). A report could contribute to multiple clusters.

### 2.5 Outcomes

We summarized openFDA outcome fields (e.g., hospitalization, life‐threatening, death). Presence of any outcome field was treated as a proxy for seriousness in descriptive summaries.

### 2.6 Signal Detection and Sensitivity Analyses

For each subcategory and reaction cluster, we computed reporting odds ratios (RORs) versus (i) the full openFDA cosmetic event database (primary) and (ii) within‐cohort comparators (broad and strict cohorts) to evaluate robustness when comparing against other facial‐skincare products [8, 9]. Confidence intervals were computed on the log scale; *p*‐values were derived from Wald tests and adjusted using FDR (*q* < 0.05) [10].

### 2.7 Case‐Check Analyses

For five prioritized signals (selected based on statistical strength and clinical interpretability), we assessed product‐name concentration (top‐1/top‐5 shares and Herfindahl–Hirschman Index), peak reporting year, sex distribution, and median age.

### 2.8 Reporting Standards

**FIGURE 1:**
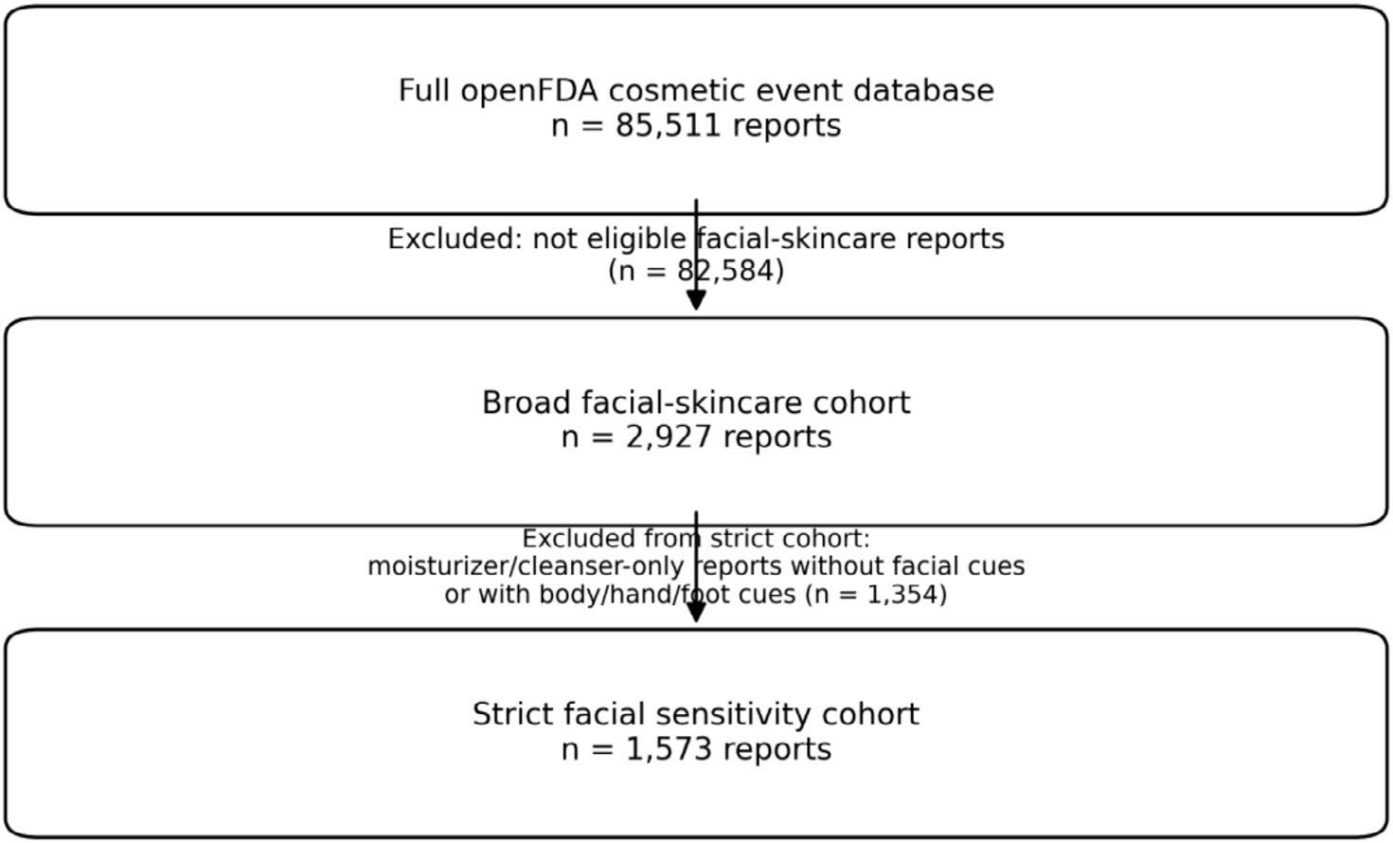
Flow diagram illustrating case selection and cohort definitions.

This study is reported in accordance with the READUS‐PV guideline for disproportionality analyses based on individual case safety reports [11, 12]. We provide a cohort selection flow diagram (Figure 1), report RORs with 95% confidence intervals for both primary and sensitivity analyses, and summarize case‐check evaluations for prioritized signals. A completed READUS‐PV checklist is provided in Table S1.

### 2.9 Ethics

The dataset is publicly available and de‐identified; no human‐subjects intervention was performed, and institutional review board approval was not required.

## 3 Results

### 3.1 Cohort Selection and Characteristics

From the full openFDA cosmetic event database (*n* = 85 511 reports), we identified a broad facial‐skincare cohort of 2927 reports by restricting to reports in which all suspect products were classified as facial‐skincare items. A strict facial sensitivity cohort (*n* = 1573) further excluded moisturizer/cleanser‐only reports without facial cues or with body/hand/foot cues (Figure 1). Key demographic and outcome characteristics are summarized in Table 1. In the broad cohort, 80.5% of reports were in females and age was available in 65.1% (median 41 years).

**TABLE 1:** Broad and strict facial‐skincare cohorts: Key characteristics.

Cohort	*N* reports	Female, *n* (%)	Age available, *n* (%)	Age, median (IQR)	Any outcome reported, *n* (%)
Broad facial cohort	2927	2355 (80.5%)	1905 (65.1%)	41.0 (28.2–55.0)	1454 (49.7%)
Strict facial cohort	1573	1337 (85.0%)	1058 (67.3%)	40.0 (29.0–54.0)	720 (45.8%)

### 3.2 Suspect Product Distribution

Moisturizers and cleansers were the most frequently implicated subcategories, appearing in 46.5% and 19.8% of reports, respectively (Table 2). Leave‐on product categories such as serum/essence (11.7%), retinoid/anti‐aging (9.9%), and masks (5.3%) accounted for a substantial minority, while toner/mist, sunscreen, and acne treatment were uncommon (< 2% each).

**TABLE 2:** Facial skincare subcategories implicated in reports (non‐mutually exclusive).

Facial skincare subcategory	Reports (*n*)	Reports (%)
Moisturizer	1362	46.5%
Cleanser	579	19.8%
Serum_essence	342	11.7%
Lip_care	319	10.9%
Retinoid_antiaging	291	9.9%
Brightening	224	7.7%
Eye_area	188	6.4%
Exfoliant_peel_scrub	172	5.9%
Mask	156	5.3%
Toner_mist	53	1.8%
Sunscreen	27	0.9%
Acne_treatment	24	0.8%

### 3.3 Adverse Event Profile

The most commonly reported MedDRA preferred terms were rash, erythema, and pruritus (each in ~16%–18% of reports), followed by burning sensation and hypersensitivity (Table 3). Overall, the reaction profile was dominated by irritation/dermatitis‐type terms and pruritic/hypersensitivity‐type terms.

**TABLE 3:** Top reaction preferred terms in the broad facial cohort (*n* = 2927).

Reaction term (MedDRA PT)	*n*	%_of_reports
Rash	520	17.8%
Erythema	507	17.3%
Pruritus	461	15.7%
Burning sensation	387	13.2%
Hypersensitivity	323	11.0%
Alopecia	257	8.8%
Pain	246	8.4%
Swelling	224	7.7%
Blister	202	6.9%
Urticaria	186	6.4%

### 3.4 Outcomes and Seriousness

**FIGURE 2:**
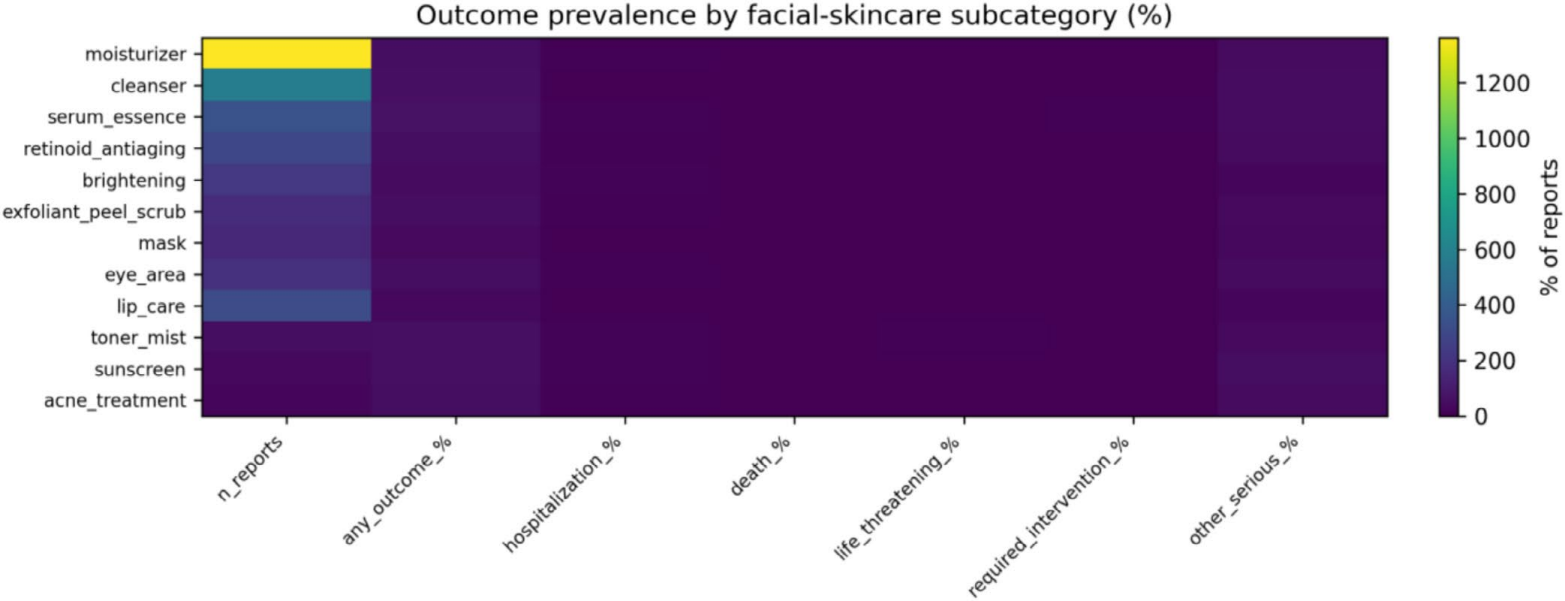
Outcome/severity heat map by subcategory (broad facial cohort).

Nearly half of reports included at least one outcome field (49.7%), most commonly “Other Serious or Important Medical Event” (39.6%) (Table 4). Hospitalization (6.3%) and death (0.5%) were uncommon overall, and the outcome/severity profile varied modestly across subcategories (Figure 2).

**TABLE 4:** Reported outcomes in the broad facial cohort (*n* = 2927).

Outcome	*n*	%_of_reports
Other serious or important medical event	1159	39.6%
Hospitalization	184	6.3%
Required intervention	78	2.7%
Life threatening	74	2.5%
Disability	65	2.2%
Death	16	0.5%
Congenital anomaly	2	0.1%

### 3.5 Temporal Trends

**FIGURE 3:**
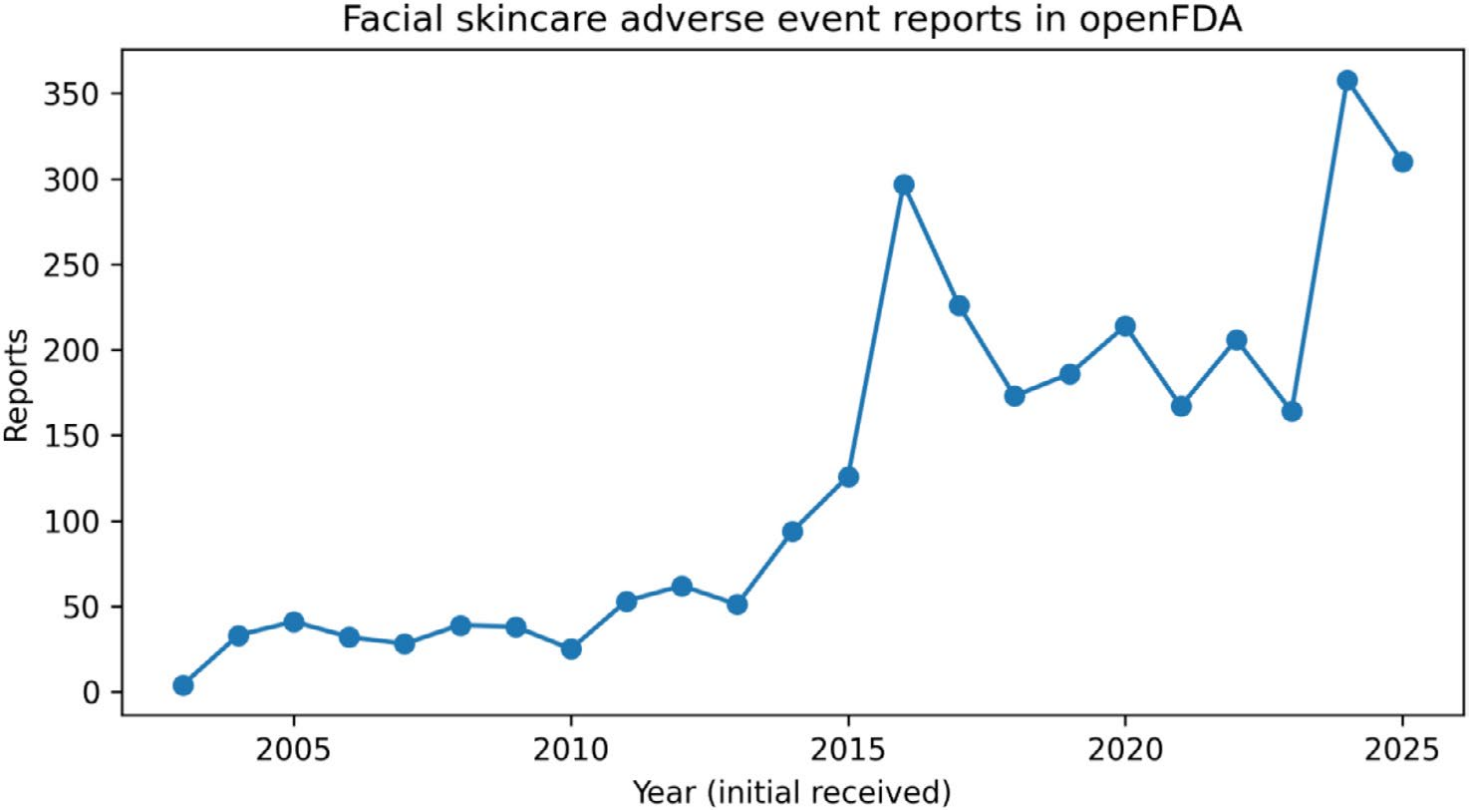
Annual reporting trend for the broad facial cohort.

Annual reporting in the broad facial cohort increased over time, with prominent increases after 2014 and peaks around 2016 and 2024 (Figure 3).

### 3.6 Disproportionality Signals Across Subcategories

**FIGURE 4:**
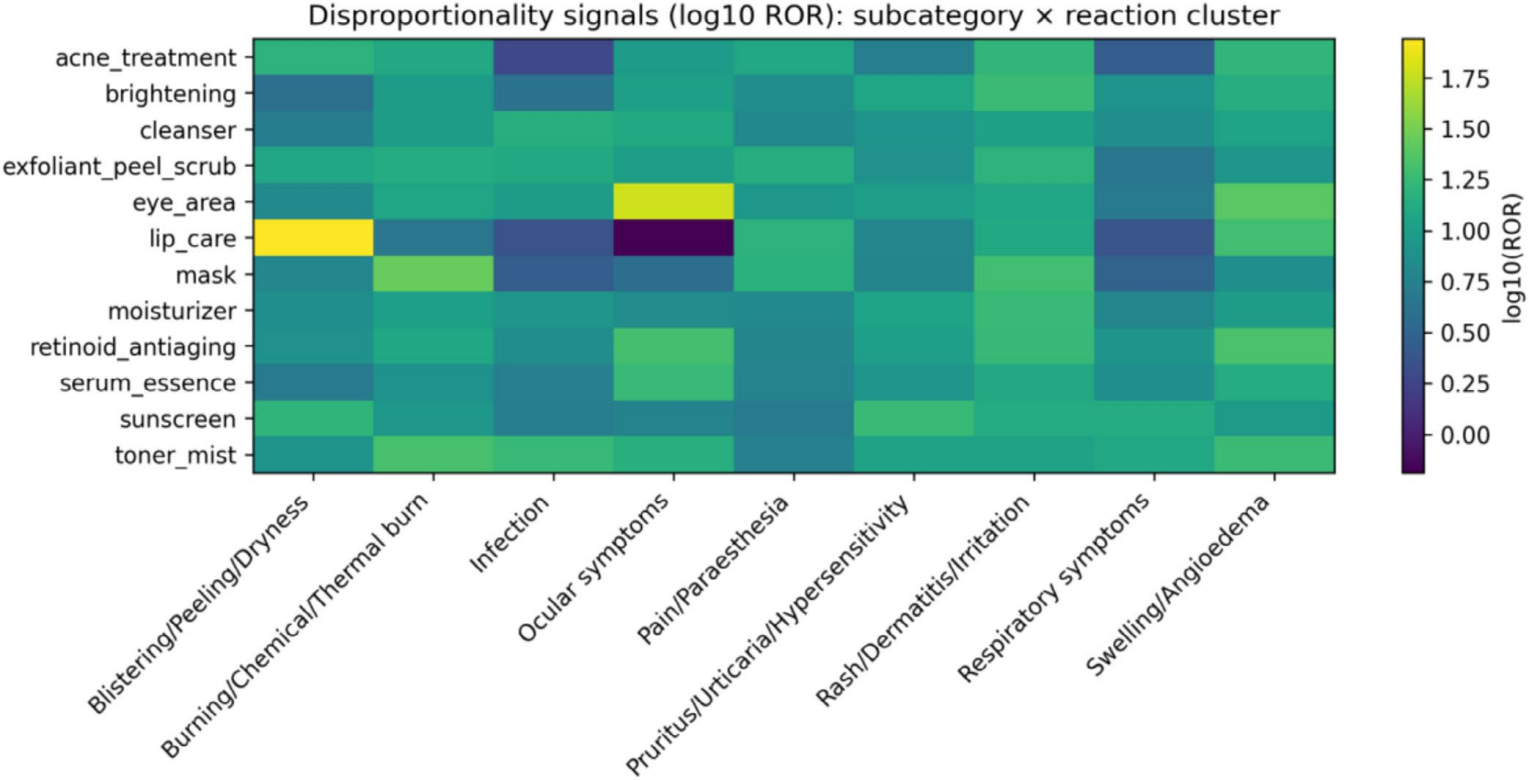
Heat map of log_10_(ROR) vs. full openFDA database (broad facial cohort).

**FIGURE 5:**
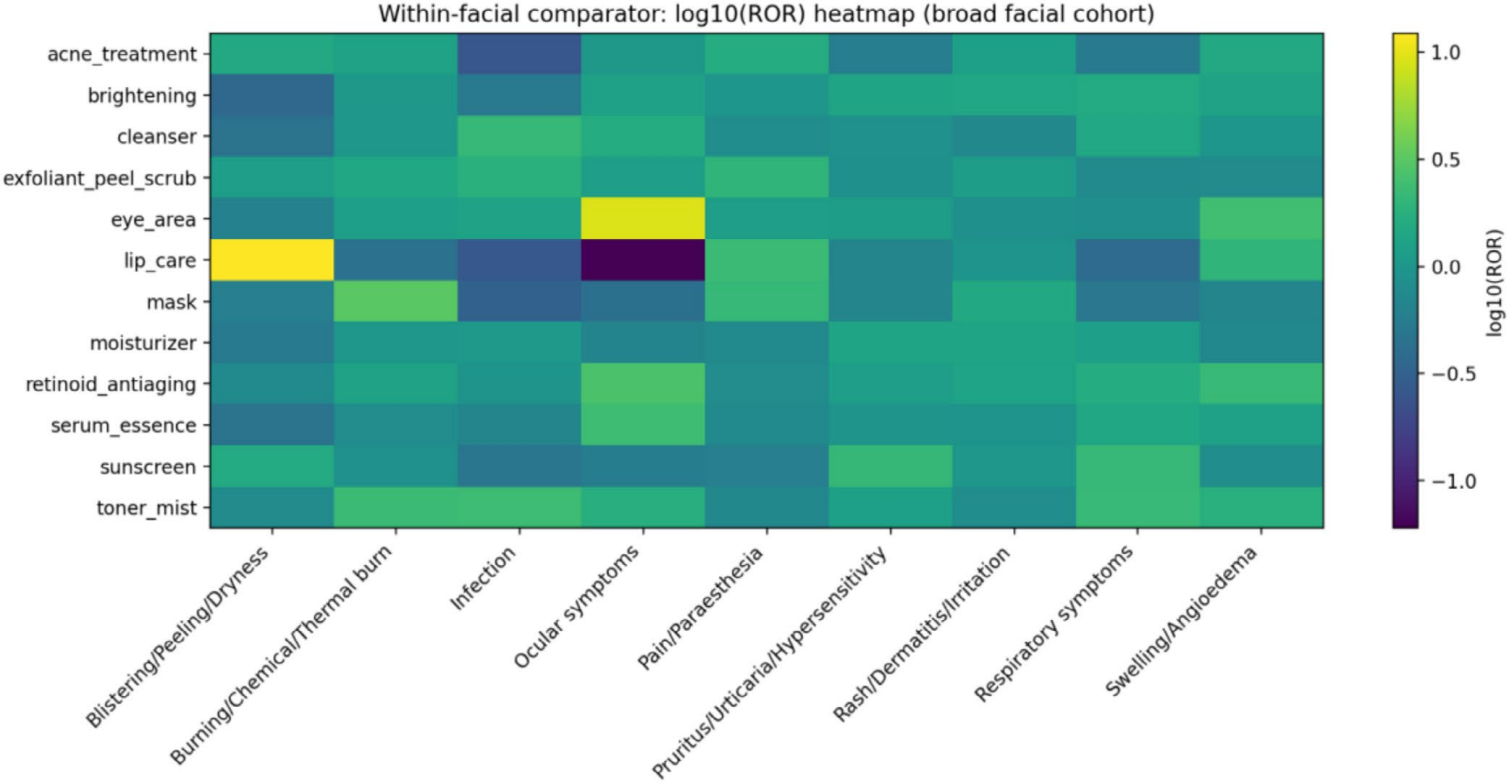
Heat map of log_10_(ROR) within the broad facial cohort (within‐cohort comparator).

Heat maps of log_10_(ROR) versus the full openFDA database highlighted several prominent category‐level signals, including eye‐area products with ocular symptoms, masks with burn‐related events, and retinoid/anti‐aging products with swelling/angioedema (Figure 4). Within‐cohort comparator analyses attenuated effect sizes but preserved these core signals, supporting robustness when comparing against other facial‐skincare products (Figure 5).

### 3.7 Within‐Cohort Prevalence

**FIGURE 6:**
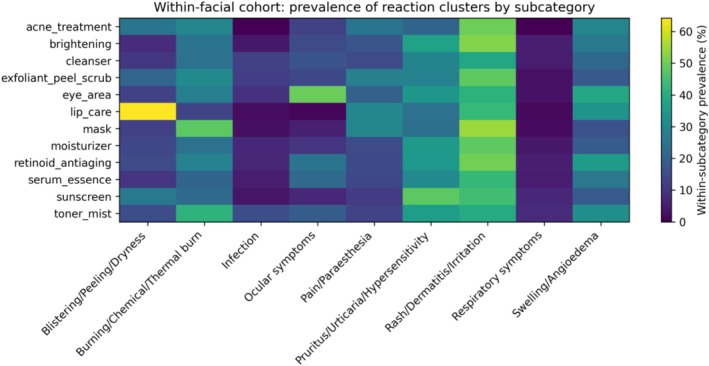
Within‐cohort prevalence heat map by subcategory and reaction cluster (broad facial cohort).

Reaction‐cluster prevalence within each subcategory mirrored the disproportionality findings, with ocular‐symptom reports most prevalent in eye‐area and serum/essence categories and burn‐related reports more prevalent in masks and exfoliant/peel/scrub categories (Figure 6).

### 3.8 Prioritized Safety Signals and Robustness

**FIGURE 7:**
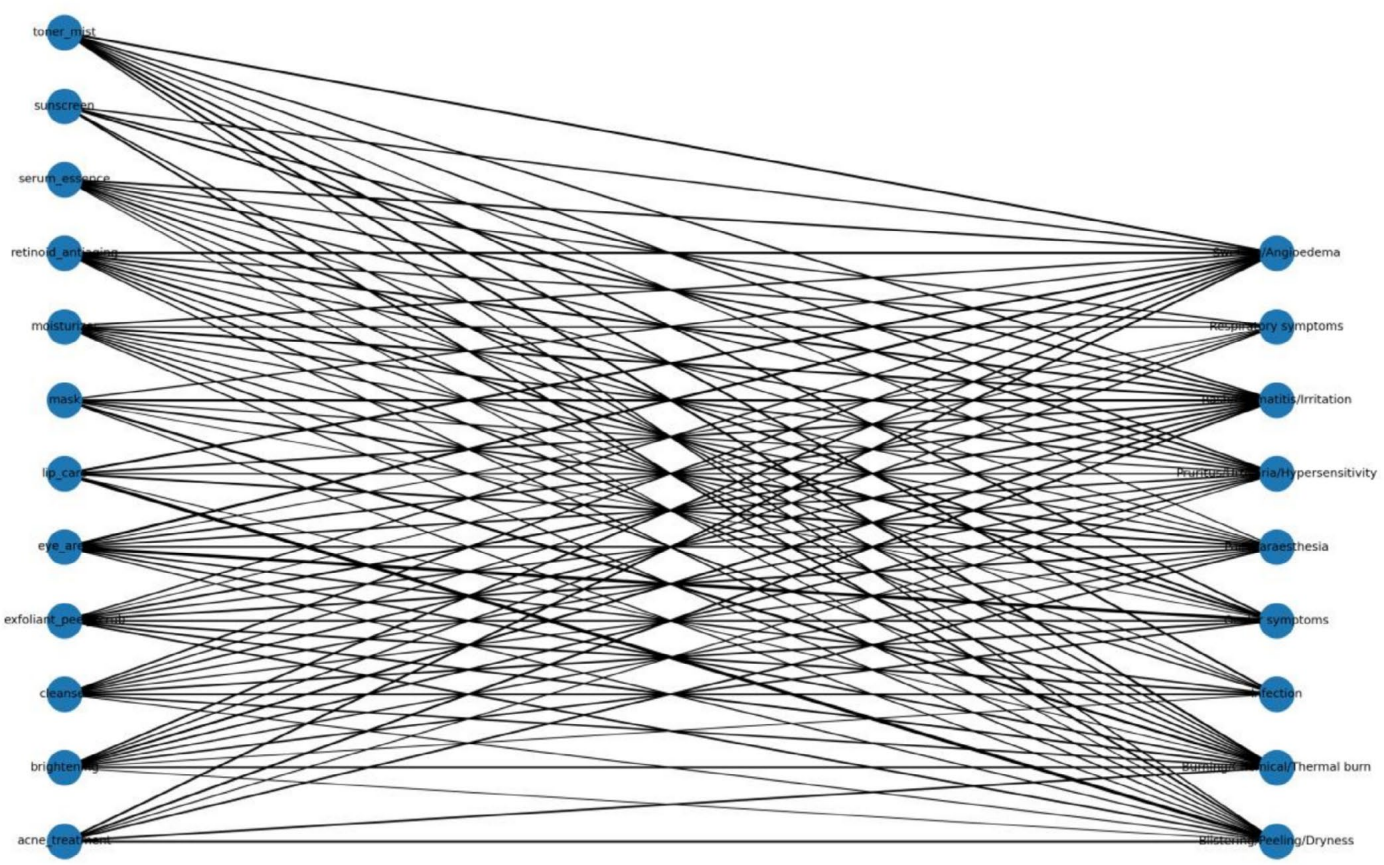
Signal network linking subcategories and reaction clusters (edge weight ~log ROR).

Table 5 summarizes five prioritized signals and their robustness to within‐cohort analyses. Four signals remained FDR‐significant within both the broad and strict cohorts, whereas the exfoliant/peel/scrub × burn‐related signal was directionally elevated but not FDR‐significant in within‐cohort comparisons. The overall signal network is visualized in Figure 7.

**TABLE 5:** Prioritized safety signals: Full‐database and within‐cohort comparator RORs.

Subcategory	Reaction cluster	Cases/exposed	Full comparator ROR (95% CI)	Within‐broad ROR (95% CI)	Within‐strict ROR (95% CI)
Eye/area	Ocular symptoms	93/188	61.67 (46.13–82.43)	9.14 (6.68–12.49)*	7.38 (5.30–10.27)*
Retinoid/antiaging	Swelling/angioedema	103/291	21.94 (17.20–28.00)	2.15 (1.66–2.79)*	1.46 (1.11–1.92)*
Mask	Burning/chemical/thermal burn	75/156	27.95 (20.39–38.31)	3.12 (2.25–4.33)*	3.37 (2.34–4.84)*
Serum/essence	Ocular symptoms	76/342	17.82 (13.74–23.10)	2.33 (1.76–3.10)*	1.61 (1.20–2.18)*
Exfoliant/peel/scrub	Burning/chemical/thermal burn	53/172	13.39 (9.68–18.53)	1.43 (1.02–1.99)	1.34 (0.93–1.94)

*Note:* Asterisks indicate FDR‐significant within‐cohort results (*q* < 0.05).

### 3.9 Case‐Check Analyses

Case‐check analyses suggested that prioritized signals were not dominated by a single product: top‐1 and top‐5 product‐name shares were low and HHI values were small across signals (Table 6), supporting category‐level interpretation. Annual case counts for each prioritized signal are shown in Figure S1 (eye‐area × ocular symptoms), Figure S2 (mask × burn‐related events), Figure S3 (retinoid/anti‐aging × swelling/angioedema), Figure S4 (serum/essence × ocular symptoms), and Figure S5 (exfoliant/peel/scrub × burn‐related events).

**TABLE 6:** Case‐check analyses for prioritized signals (product concentration and temporal patterns).

Signal	Reports (a)	Unique products	Top‐1 share	Top‐5 share	HHI	Female %	Median age (years)	Peak year (reports)
Eye/area × Ocular symptoms	93	90	6.1%	13.3%	0.014	90.3%	47.5	2015 (11)
Mask × Burn‐related	75	73	3.8%	12.8%	0.015	96.0%	31.0	2020 (20)
Retinoid/antiaging × Swelling/Angioedema	103	92	3.9%	13.6%	0.013	98.1%	54.5	2024 (13)
Serum/essence × Ocular symptoms	76	76	3.8%	11.2%	0.014	92.1%	51.5	2024 (13)
Exfoliant/peel/scrub × Burn‐related	53	56	1.8%	8.9%	0.018	84.9%	38.5	2018 (7)

### 3.10 Prioritized Safety Signals

Table 5 summarizes five prioritized signals and their robustness to within‐cohort comparator analyses.

### 3.11 Case‐Check Analyses

Table 6 summarizes product concentration and temporal patterns for prioritized signals.

## 4 Discussion

Using openFDA cosmetic adverse event reports, we mapped facial‐skincare reporting patterns and identified multiple strong, clinically coherent disproportionality signals. The strongest signals involved eye‐area products and ocular symptoms, consistent with the sensitive periocular region and the potential for product migration or accidental ocular exposure [13]. Signals for burn‐like events were prominent for masks and exfoliant/peel/scrub products, which may reflect irritant potential, misuse, or higher‐risk formulations [14]. Retinoid/anti‐aging products showed strong swelling/angioedema signals, consistent with hypersensitivity‐type reports in susceptible individuals.

Sensitivity analyses supported robustness of key findings. Within‐cohort comparator RORs attenuated relative effect sizes (as expected when comparing against other facial‐skincare products) but preserved core signals (eye‐area × ocular symptoms; mask × burn‐related events; retinoid/anti‐aging × swelling/angioedema; serum/essence × ocular symptoms). The exfoliant/peel/scrub × burn signal remained directionally elevated but did not survive FDR in within‐cohort analyses, suggesting that burn‐related reports are shared across multiple leave‐on facial product categories.

Case‐check analyses suggested that prioritized signals were not dominated by a single product or a small number of products. Instead, product mentions were distributed across many unique product names with low concentration metrics, supporting a category‐level signal interpretation.

### 4.1 Limitations

Spontaneous reports are subject to under‐reporting, stimulated reporting, missing data, and confounding (including concomitant products and underlying skin conditions) [6, 9]. Product classification relied on product‐name keywords and may misclassify some multi‐use products. Disproportionality metrics identify reporting patterns and generate hypotheses; they do not establish causality [9].

## 5 Conclusion

Publicly available openFDA cosmetovigilance data can be leveraged to map facial‐skincare adverse event reporting patterns and to prioritize potential safety signals. Eye‐area products showed particularly strong ocular‐related signals, and leave‐on products (masks, retinoid/anti‐aging, serum/essence) showed coherent burn‐ and swelling‐related signals. These findings may inform clinical counseling and post‐market safety monitoring.

## Funding

This work was supported by the Shaanxi Provincial Natural Science Basic Research Program—General Project (2024JC‐YBMS‐633).

## Conflicts of Interest

The authors declare no conflicts of interest.

## Supporting information


**Table S1:** READUS‐PV checklist (completed).
**Figure S1:** Eye‐area × ocular symptoms: annual case counts.
**Figure S2:** Mask × burn‐related events: annual case counts.
**Figure S3:** Retinoid/anti‐aging × swelling/angioedema: annual case counts.
**Figure S4:** Serum/essence × ocular symptoms: annual case counts.
**Figure S5:** Exfoliant/peel/scrub × burn‐related events: annual case counts.

## Data Availability

The data analyzed in this study are publicly available from the US FDA openFDA Cosmetic Adverse Events database (bulk) at https://open.fda.gov/apis/cosmetic/event/download/ [4]. Code availability: Analyses were performed in Python (v3.11). The code used for product keyword classification, reaction clustering, and signal detection is available from the corresponding author upon reasonable request.
