# Supplementary material for: Facial Skincare Adverse Event Atlas and Safety Signals From openFDA Cosmetic Reports: A Disproportionality Analysis
Source: J Cosmet Dermatol. 2026 Jan 30;25(2):e70712. doi: 10.1111/jocd.70712 (PMC12859173; doi:10.1111/jocd.70712)

**SUPPLEMENTARY TABLES**

*Table S1. READUS-PV checklist (completed)*

| Item | Checklist item (paraphrased) | Location in revised manuscript |
| --- | --- | --- |
| 1a | Identify the study as a disproportionality analysis; specify data type and database name. | Title |
| 1b | Name the adverse event(s) and/or product(s) under study, when applicable. | Title; Abstract (Objective/Results) |
| 2a | Describe the product(s) and utilization, and the nature/frequency of adverse events and existing knowledge. | Introduction |
| 2b | State the rationale for performing the analysis (routine surveillance, safety profile, hypothesis). | Introduction |
| 2c | Explain why ICSR databases and disproportionality analysis are suitable for the knowledge gap. | Introduction; Methods (Data source and study design) |
| 3 | State specific objectives, including adverse events, products, reference group, and any pre-specified hypothesis. | Abstract (Objective); Methods (Signal detection) |
| 4a | Identify the study as a disproportionality analysis and specify the type of data (ICSRs). | Methods (Data source and study design; Reporting standards) |
| 4b | Outline the full study design, including primary and sensitivity analyses and any case-by-case analysis. | Methods (Signal detection; Case-check analyses; Reporting standards) |
| 5a | Describe the database, custodian, and coverage; specify coding systems/taxonomies. | Methods (Data source and study design; Database characteristics) |
| 5b | Specify extraction date and all data pre-processing/transformation/exclusion choices. | Methods (Data source and study design; Cohort definition) |
| 6a | Describe the study population and any restriction. | Methods (Cohort definition); Results (Cohort selection) |
| 6b | Describe key variables and their meaning. | Methods (Cohort definition; Reaction clustering; Outcomes) |
| 6c | Specify/justify grouping of products/events and selected product role. | Methods (Cohort definition; Reaction clustering) |
| 6d | Describe any additional data sources and how they interact with ICSRs. | Not applicable (no additional data sources used) |
| 7a | Describe descriptive analyses, tests, and significance thresholds. | Methods (Outcomes; Signal detection) |
| 7b | Describe the disproportionality measure(s) and signal thresholds. | Methods (Signal detection); Table 5; Figures 3–4 |
| 7c | Describe sensitivity analyses and approaches to control confounding. | Methods (Signal detection); Figures 4–5; Table 5 |
| 7d | Specify variables/methods used for any case-by-case analysis (incl causality). | Methods (Case-check analyses); Table 6 |
| 7e | Specify statistical methods used for other data sources. | Not applicable |
| 8a | Report the number of reports included at each stage, with reasons for exclusion. | Results (Cohort selection); Figure 1 |
| 8b | Provide key demographic/clinical characteristics (and comparator if relevant). | Table 1; Results (Cohort selection) |
| 9 | Present all results with confidence intervals and sensitivity analyses. | Table 5; Figures 3–4; Results |
| 10 | Present case-by-case analysis results and causality assessment, if applicable. | Case-check analyses presented in Table 6 (no formal causality assessment performed) |
| 11 | Discuss key results in context and distinguish expected reactions vs emerging signals. | Discussion |
| 12a | Discuss external validity to the general population. | Discussion; Limitations |
| 12b | Discuss potential relevance in clinical practice. | Discussion; Conclusion |
| 12c | Propose further study designs, if applicable. | Discussion (Limitations/Future work) |
| 13 | State limitations (reporting bias, confounding, non-causality, no incidence). | Discussion (Limitations paragraph) |
| 14a | Funding/sponsorship and role of funders. | Funding |
| 14b | Conflicts of interest. | Conflict of interest |
| 14c | Institutional approval/ethics. | Ethics |
| 14d | Data availability, code availability (including software version), and protocol registration. | Data availability (Data + Code availability paragraphs) |

**SUPPLEMENTARY FIGURES**

*Figure S1. Eye-area × ocular symptoms: annual case counts.*


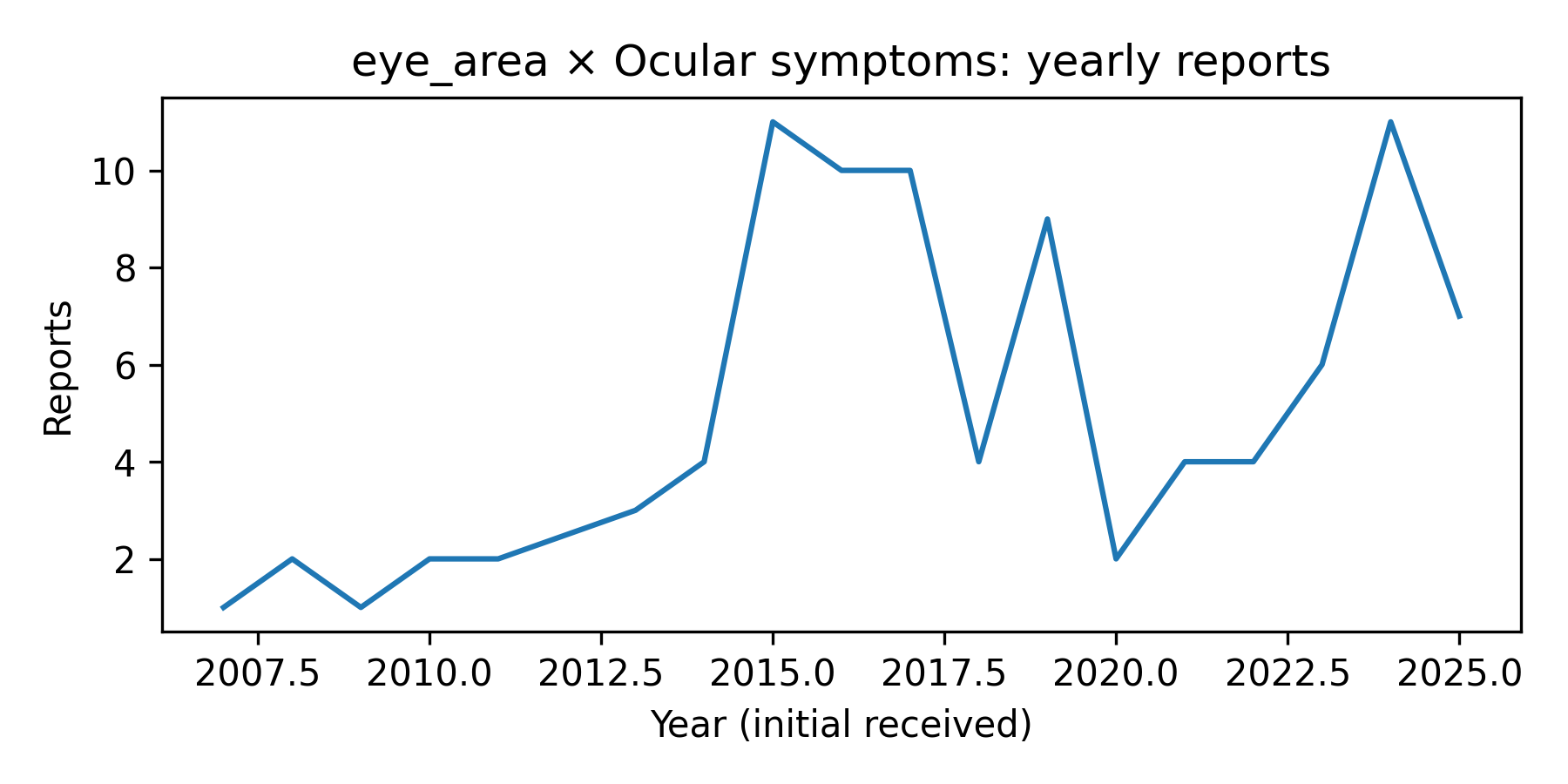


*Figure S2. Mask × burn-related events: annual case counts.*


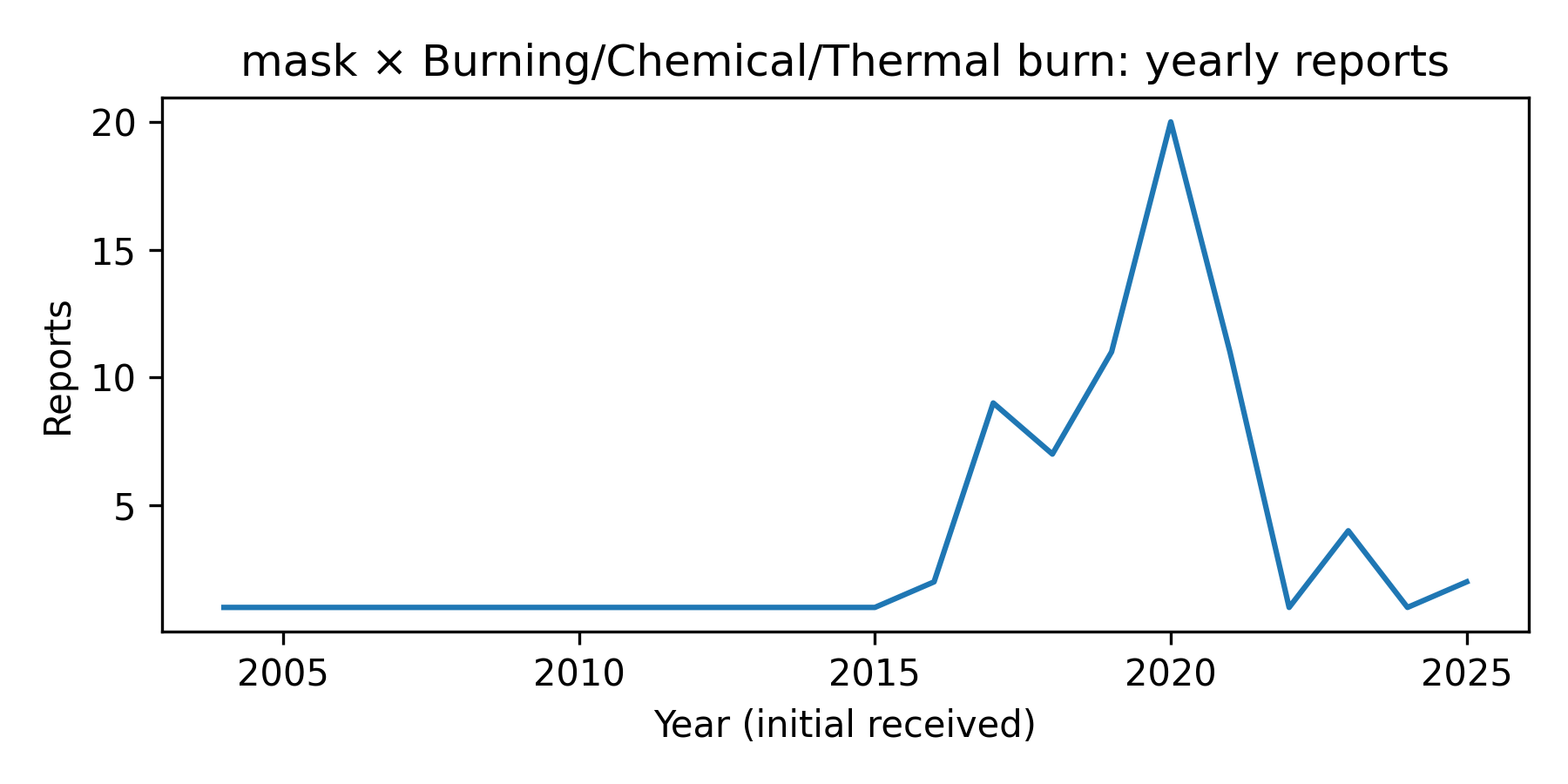


*Figure S3. Retinoid/anti-aging × swelling/angioedema: annual case counts.*


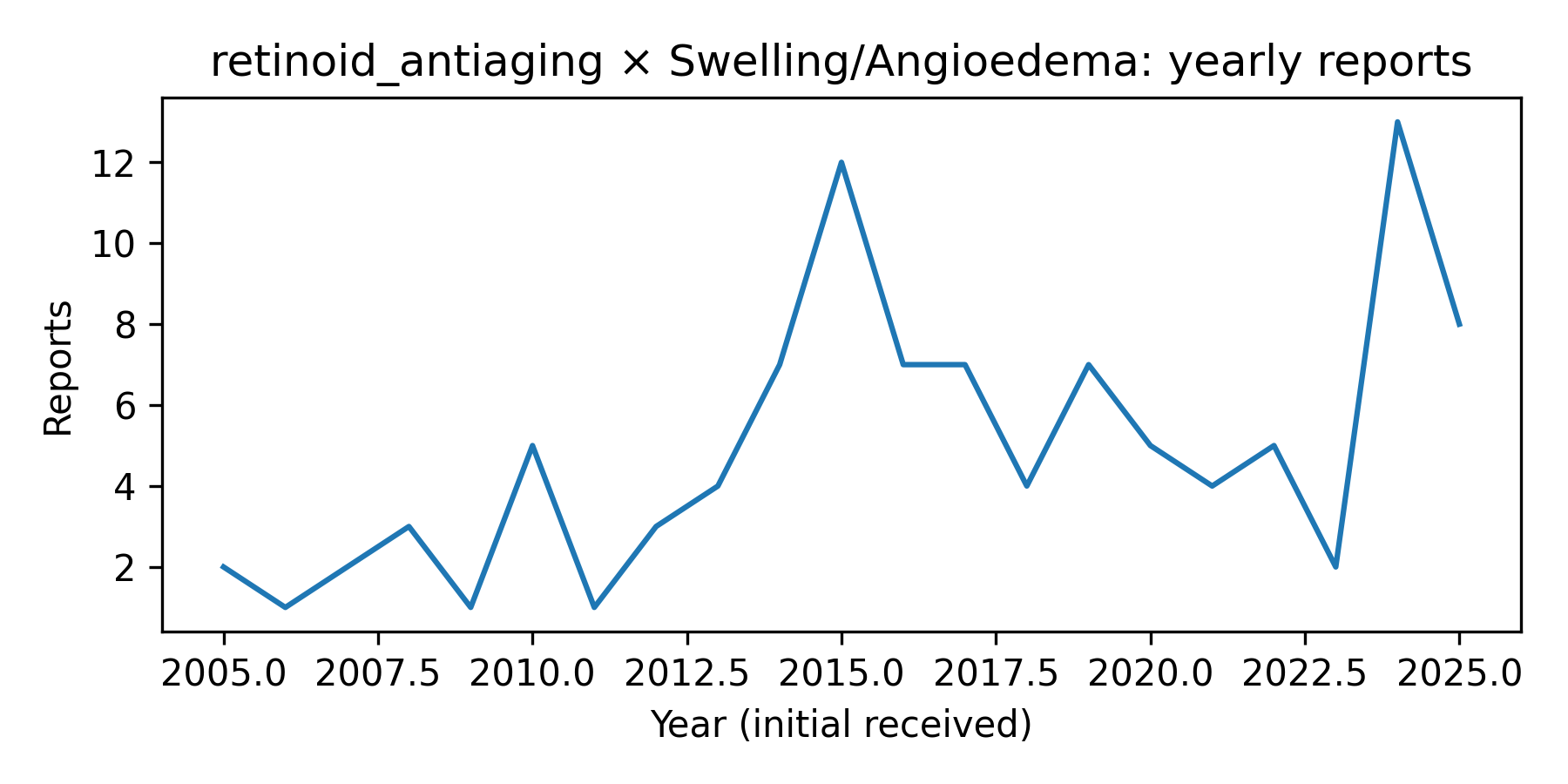


*Figure S4. Serum/essence × ocular symptoms: annual case counts.*


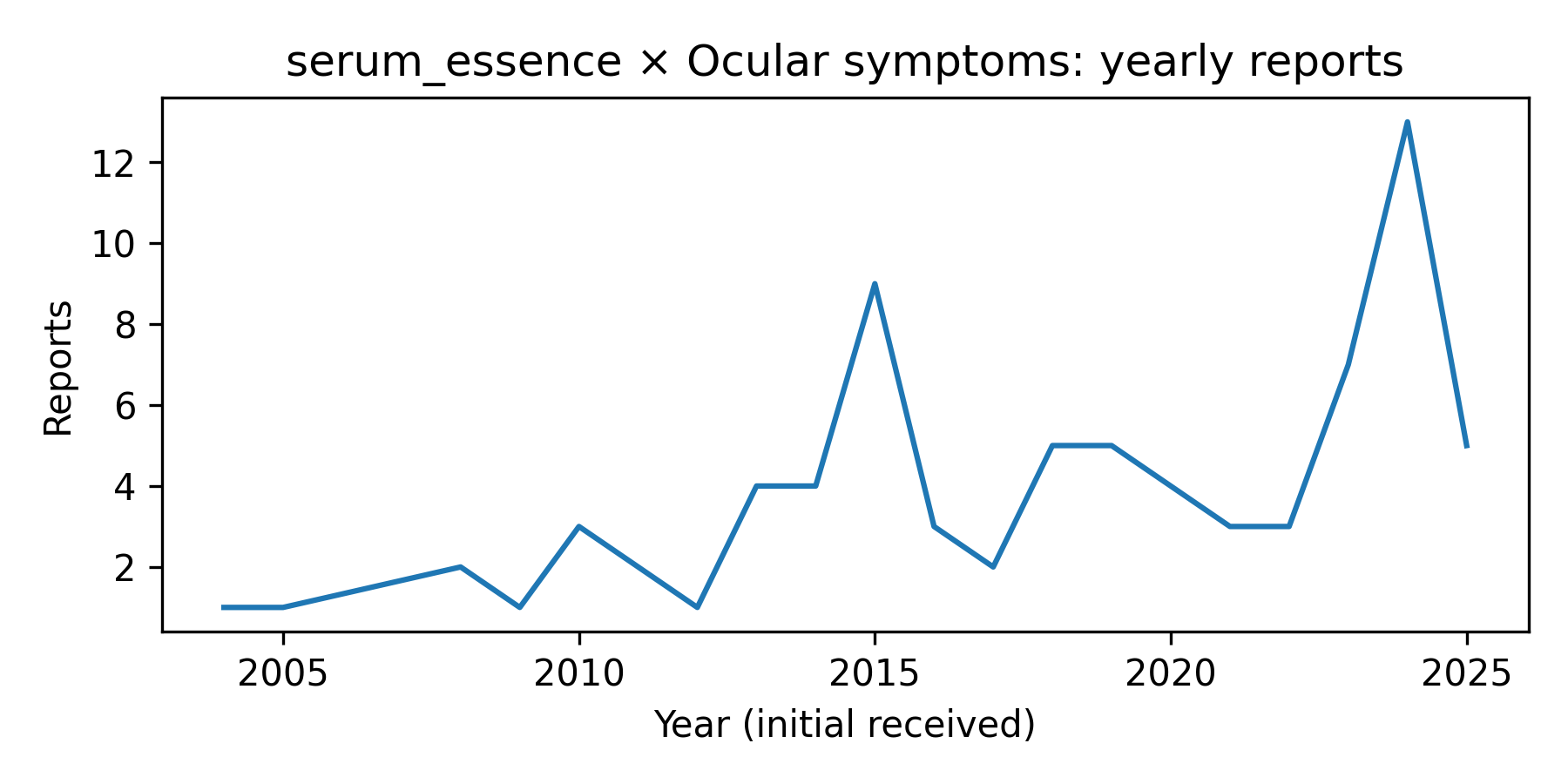


*Figure S5. Exfoliant/peel/scrub × burn-related events: annual case counts.*


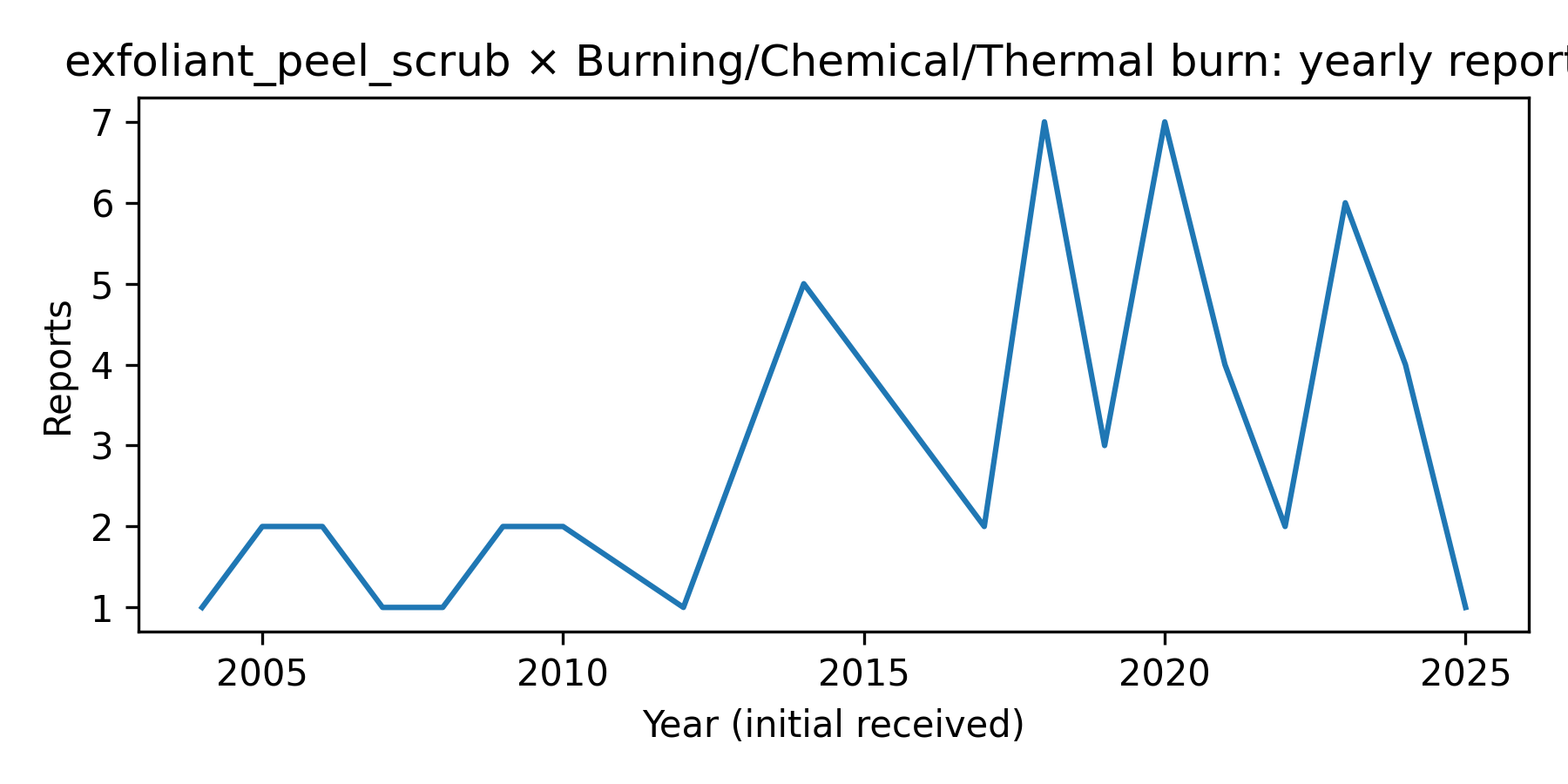

Supplement: Supplementary file 1 — Table S1: READUS‐PV checklist (completed). Figure S1: Eye‐area × ocular symptoms: annual case counts. Figure S2: Mask × burn‐related events: annual case counts. Figure S3: Retinoid/anti‐aging × swelling/angioedema: annual case counts. Figure S4: Serum/essence × ocular symptoms: annual case counts. Figure S5: Exfoliant/peel/scrub × burn‐related events: annual case counts. [file JOCD-25-e70712-s001.docx]
